# Integrated transmission assessment surveys (iTAS) of lymphatic filariasis and onchocerciasis in Cross River, Taraba and Yobe States, Nigeria

**DOI:** 10.1186/s13071-022-05302-x

**Published:** 2022-06-13

**Authors:** Ifeoma N. Anagbogu, Yisa A. Saka, Olabanji Ahmed Surakat, Chukwu Okoronkwo, Emmanuel Davies, Philip Oyale, Uwem Friday Ekpo, Uche V. Amazigo, Kira Barbre, Micheal Igbe, Audrey Nyior, Solomon M Jacob, Uduak Gideon Nteun, Zaiyanatu Abubakar Umar

**Affiliations:** 1 Federal Ministry of Health, Abuja, Nigeria; 2 Federal Ministry of Health, Abuja, Nigeria; 3 Department of Zoology, Faculty of Basic and Applied Sciences, Osun State University, Osogbo, Nigeria; 4 Department of Pure & Applied Zoology, Federal University of Agriculture, Abeokuta, Nigeria; 5 African Programme for Onchocerciasis Control, Ouagadougou, Burkina Faso; 6 Neglected Tropical Diseases Support Center, The Task Force for Global Health, Atlanta, GA USA

**Keywords:** Local government areas, Integrated transmission assessment survey, Pre-TAS, Mass drug administration, Onchocerciasis, Lymphatic filariasis, Nigeria

## Abstract

**Background:**

Integrated transmission assessment surveys (iTAS) have been recommended for evaluation of the transmission of both lymphatic filariasis (LF) and onchocerciasis as the prevalence of both diseases moves toward their respective elimination targets in Nigeria. Therefore, we conducted an iTAS between May and December 2017 in five local government areas (LGAs), also known as implementation units (IUs), in states of Cross River, Taraba and Yobe in Nigeria.

**Methods:**

The TAS comprised two phases: the Pre-iTAS and the iTAS itself. Three states (Cross River, Taraba and Yobe), comprising five LGAs and 20 communities that have completed five rounds of combined treatment with ivermectin and albendazole for LF and 12 total rounds of ivermectin, were selected for inclusion in the study. All participants were tested with the Filariasis Test Strip (FTS; Alere Inc.) and the Biplex rapid Diagnostic Test (RDT; identifying filaria antigens Ov16/Wb123; Abbott diagnosctics Korea Inc.). Pre iTAS included 100 children ages 5-9 in each 4 communities and 300 individuals ages 10 and older in a subset of two communities.  For the iTAS, only LGAs where antigenemia prevalence in all sampled communities during the Pre-iTAS was < 2% for LF were selected.

**Results:**

Of the five LGAs included in the study, four met the cutoff of the Pre-iTAS and were included in the iTAS; the Ikom LGA was excluded from the iTAS due to antigenemia prevalence. A total of 11,531 school-aged children from 148 schools were tested for LF and onchocerciasis across these four LGAs, including 2873 children in Bade, 2622 children in Bekwara, 3026 children in Gashaka and 3010 children in Karim Lamido. Using the FTS, all samples from Bade and Karim Lamido were negative, whereas 0.2% of the samples from Bekwara and Gashaka were positive. Using the Biplex RDT, LF prevalence in Bade, Bekwara, Gashaka and Karim Lamido was < 0.1%, 0.5%, 0.4% and < 0.1%, respectively. Moreover, all samples from Bade and Karim Lamido were negative for onchocerciasis, whereas 3.1% and 1.8% of the samples from Bekwara and Gashaka were positive, respectively.

**Conclusion:**

This study has provided additional information on the current burden of onchocerciasis and LF in the four IUs sampled where mass drug administration (MDA) for both infections has been ongoing for years. The study identifies that LF-MDA can be safely stopped in all four of the IUs studied, but that MDA for onchocerciasis needs to continue, even though this may pose a challenge for LF surveillance. Based on the preliminary results from all four sites, this study has fulfilled the primary objective of determining the programmatic feasibility of an iTAS as a tool to simultaneously assess onchocerciasis and LF prevalence in areas co-endemic for the two infections that have completed the recommended treatment for one or both infections, and to make decisions on how to proceed.

**Graphical Abstract:**

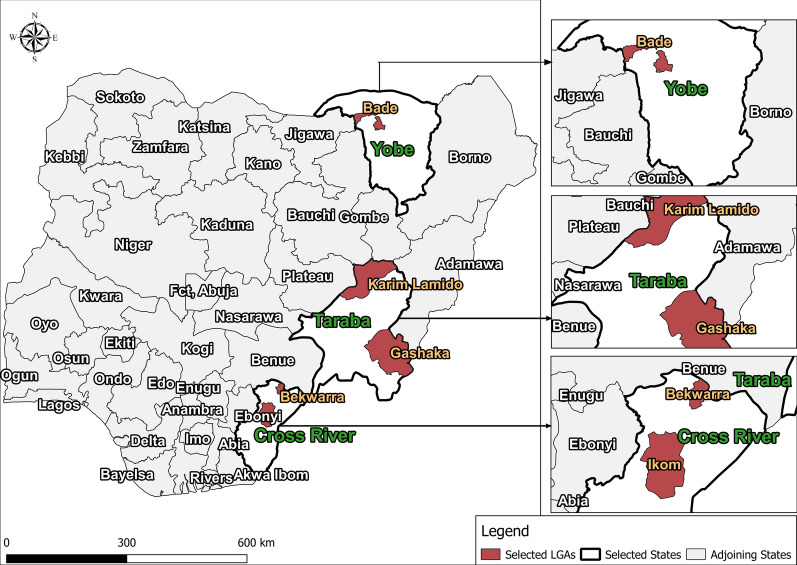

## Background

Lymphatic filariasis (LF) and onchocerciasis are infections caused by the microscopic nematodes *Wuchereria bancrofti* and *Onchocerca volvulus* transmitted respectively by mosquitoes and black flies of the genus *Simulium* [1, 2]. LF has been found to be endemic in 73 countries, with 1.1 billion individuals projected to be at risk, and at least 169 million people in 31 countries in Africa are estimated to be at risk of infection with onchocerciasis and to require mass drug administration (MDA) [3].

The control strategy for LF in Africa is MDA of single-dose combination therapy with albendazole and ivermectin or diethylcarbamazine [4]. When given for an estimated period of 5–6 years with an annual minimum therapeutic coverage of 65%, MDA can interrupt the transmission of *W. bancrofti* in a population. Onchocerciasis control and elimination efforts focus on MDA using ivermectin, with sporadic vector control where conditions are favourable [5, 6]. When treatment occurs at least once a year for about 15 years or more, it is possible to interrupt transmission [7].

A total of 583 local government areas (LGAs) are endemic for LF in Nigeria; of these, 567 (97%) have conducted at least one MDA round, with 30 of these 567 LGAs also under post-MDA surveillance. Sixteen LGAs have yet to start MDA due to funding challenges [5]. About 50 million persons in over 40000 communities in Nigeria are at risk of onchocerciasis infection, accounting for about 40% of the global population at risk. A total of 480 LGAs are targeted for the elimination of onchocerciasis transmission with > 85% geographic coverage currently being achieved [8].

The African Programme for Onchocerciasis Control (APOC), which replaced the Onchocerciasis Control Programme (OCP), supported the control of onchocerciasis through annual or 6-monthly ivermectin-based MDA as well as vector control in selected foci [9]. APOC goals transitioned from the control to the elimination of onchocerciasis after a study that was conducted in Mali and Senegal was published in 2009, which provided the first evidence that the elimination of onchocerciasis was feasible in Africa with ivermectin distribution alone [10]; APOC then developed a framework to guide countries towards elimination [2]. By 2012, the transition from morbidity control to elimination of the transmission of onchocerciasis infection was officially documented in the WHO road map on neglected tropical diseases [11]. In 2016, WHO published guidelines on the criteria for stopping MDA in areas that have completed at least 12–15 years of treatment at a minimum of 80% coverage [4]. One of the conditions for stopping MDA is antibody prevalence of < 0.1% in children aged < 10 years.

National LF programmes focus on a series of critical steps following standardized WHO protocols: (i) mapping to determine endemicity; (ii) implementation of MDA with a minimum of 65% treatment coverage once yearly for at least 5 years in endemic areas; (iii) a survey to determine whether prevalence is low enough for MDA to stop; (iv) post-MDA surveillance; and (v) validation of elimination of LF as a public health problem.

There is substantial overlap between LF and onchocerciasis in Nigeria, with both diseases overlapping in 366 LGAs. In those co-endemic LGAs, ivermectin is distributed with albendazole during MDA campaigns. Filariasis programmes in countries have typically functioned vertically and, until a few years ago, in Nigeria, the decision to start MDA was often made for each disease independently without any consideration of the endemicity of other disease.

As Nigeria begins to reach a sufficient number of effective rounds of MDA for both LF and onchocerciasis, a decision needs to be made on whether an implementation unit (IU) or an evaluation unit (EU) can safely stop MDA for one infection without compromising the success of both elimination programmes. The addition of antibody testing for onchocerciasis into the LF transmission assessment survey (TAS), therefore, could be a cost-effective tool to determine whether additional rounds of MDA with ivermectin might be needed. If the results indicate the presence of ongoing transmission of either LF or onchocerciasis among young children, this will provide sufficient evidence to continue ivermectin MDA. If the results do not indicate ongoing transmission of either infection, however, further investigations, including entomological assessments, will be needed to determine whether it is safe to stop ivermectin MDA for onchocerciasis [4]. Therefore, it may be possible to integrate a serologic study that meets the serologic component for stopping MDA for onchocerciasis into a routine LF TAS.

Based on this background, the aim of this study was to determine the programmatic feasibility of an integrated LF transmission assessment survey (iTAS) and onchocerciasis epidemiological evaluation for simultaneous assessment of both diseases in potentially co-endemic areas that have completed the recommended period of treatment for both diseases.

## Methods

### Study sites

Five LGAs (IUs), namely Ikom and Bekwara, Gashaka and Karim-Lamido and Bade, were selected for inclusion in the Pre-iTAS. These LGAs were selected based on their geographic location across different ecological zones and have successfully conducted a minimum of five effective rounds of ivermectin and albendazole combined treatment for LF, as well as > 12 effective rounds of ivermectin treatment alone for onchocerciasis. The last MDA prior to the present study for both infections was conducted from August through to November 2016. Bekwara and Ikom are located in Cross River State, in the South-South geo-political zone, within the tropical rain forest; they are characterized by their flat landscape. Gashaka and Karim Lamido are located in Taraba State, in the North-East geo-political zone, and fall largely within the guinea savanna belt, with undulating landscape dotted with a few mountainous features. The vegetation comprises low forest in the southern part and grassland in the northern part. Bade is located in Yobe state, also in the North-East geo-political zone, but in the arid Sudan Savanah belt.

**Fig. 1:**
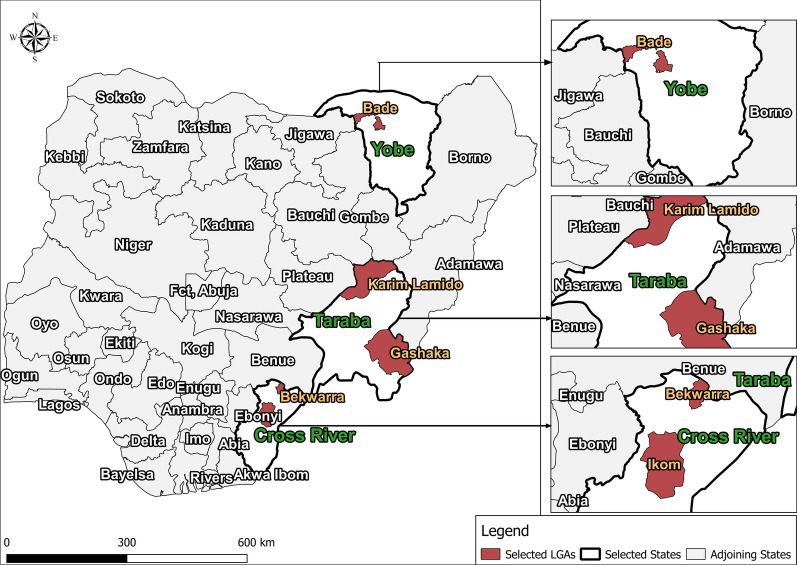
Map of Nigeria showing the study areas. Abbreviations: LGA, Local government areas

**Fig. 2:**
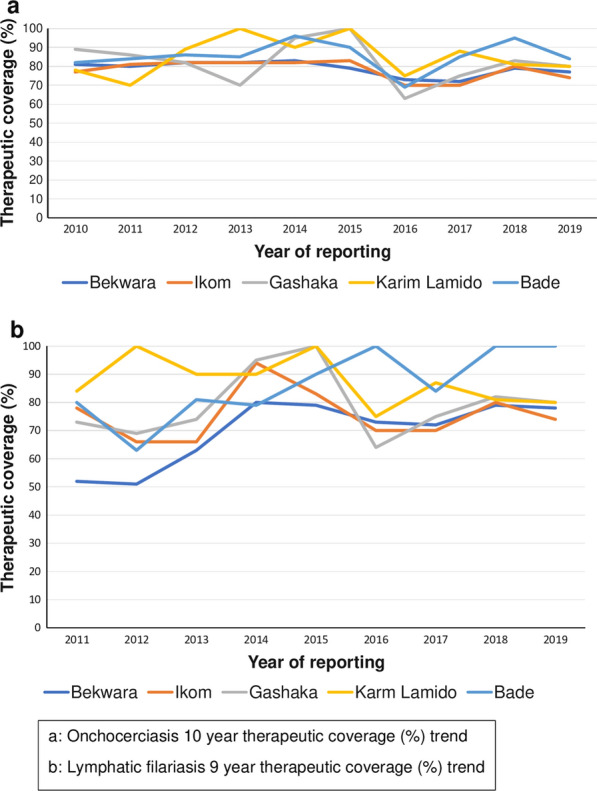
Treatment coverage in the selected LGAs 2011–2019. **a** Onchocerciasis 10-year trend in therapeutic coverage (%), **b** lymphatic filariasis 9-year trend in therapeutic coverage (%)

The location of the study LGAs is shown in Fig. 1. Baseline information, including prevalence (nodule for onchocerciasis and antigenemia for LF) is given in Table 1. Coverage in onchocerciasis MDA (2011–2019) has been > 70% in all five selected LGAs except for slight dips in 2016 (Fig. 2). Coverage in LF MDA has been > 65% except for Bekwerra, which had low treatment in 2011 through 2013. Data quality issues occur regarding the coverage in some years for the various LGAs in both the onchocerciasis and LF MDA campaigns.

**Table 1:** Baseline prevalence of Onchocerciasis and lymphatic filariasis in the study areas

State	LGA (IU)	Onchocerciasis				Lymphatic filariasis			
		Year of mapping	Method	Range in baseline prevalence, %	Year MDA started	Year of mapping	Method	Baseline prevalence (%)	Year MDA Started
Cross River	Bekwara	1989	REMO	7.6–24	1994	2010	ICT	1	2011
	Ikom	1998	REMO	14.8–62.5	1994	2009	ICT	2	2010
Taraba	Gashaka	1998	REMO	5.4–65.5	1992	2008	ICT	8	2009
	Karim Lamido	1998	REMO	2.7–24.2	1997	2008	ICT	6.7	2009
Yobe	Bade	2000	REMO	7.2–20.6	1995	2008	ICT	1.3	2010

*ICT* Immunochromatographic test, *IU* implementation unit,* LGA* local government area,* MDA* mass drug administration,* REMO* rapid epidemiological mapping of onchocerciasis

### Study design, selection of study sites and sampling strategies

The study was divided into two phases: the Pre-iTAS and the main iTAS. The initial phase (Pre-iTAS) was conducted in the five LGAs in April 2017, in accordance with WHO criteria for conducting LF TAS [14]. All LGAs that met the cutoff in the pre-iTAS were included in the iTAS, which was conducted from May through June 2017.

#### Pre-iTAS

In each selected LGA (IU), two sites were selected to meet the criteria of an LF pre-TAS. This included one LF sentinel site with a high baseline prevalence for LF and one onchocerciasis sentinel site based on a high risk of onchocerciasis infection according to the baseline rapid epidemiological mapping of onchocerciasis (REMO) data. The onchocerciasis sentinel site served as a spot-check site for LF. In each of these two sentinel sites per IU, a total of 300 older children and adults of all ages  ≥ 10 years were selected by convenience. They  were tested for both LF and onchocerciasis using the Filariasis Test Strip (FTS; Alere Inc., Waltham, MA, USA) and the SD BIOLINE Oncho/LF IgG4 Biplex Rapid Diagnostic Test (RDT; identifying filarial antigens Ov16/Wb123; Abbott Diagnostics Korea Inc. Giheung-gu, Republic of Korea).

For the onchocerciasis assessment, two additional first-line communities (sites) were selected in each LGA (IU) in addition to the LF sentinel site and spot-check site as described above (Table 2). Both additional selected communities had a high risk of onchocerciasis infection according to baseline REMO data. Both were first-line communities (with no settlement between them and pre-identified potential breeding sites). Those persons who were not included in the REMO baseline database were within a radius of 50 km of a baseline REMO site. A total of 100 children aged 5–9 years were sampled in each of the four communities through convenience sampling and tested for both LF and onchocerciasis using FTS and Biplex RDT (Ov16/Wb123).

**Table 2:** Communities selected for inclusion in the Pre-ITAS conducted in the local government areas/implementation units

State	LGA (IU)	Communities
Cross River	Bekwara	Afrike
		Itekpa
		Atibulum
		NyanaOlim
	Ikom	Agbaragba
		Balep
		Etikpe
		Ukpochi
Taraba	Gashaka	Gashaka
		Mayo Selbe
		Nybar
		Shinbon
	Karim Lamido	Bandawa
		Gwomu
		Kodei
		Panya
Yobe	Bade	Dagona
		Gafiwa
		Gwio Kura
		Tagali

*Pre-ITAS* Pre-integrated transmission assessment survey

#### iTAS

The qualification for inclusion in the iTAS was that all the selected sites in an LGA (IU) achieve < 2% antigenemia prevalence for LF. Any LGA with at least one selected community that had an antigenemia prevalence > 2% during pre-iTAS would therefore not progress to/be included in iTAS. Using systematic random cluster sampling to give an equal chance of selection for each school and eliminate bias, a minimum of 30 schools were selected from each LGA from a sampling frame of all the primary schools in the LGA. In order to fulfill the current stopping MDA threshold for onchocerciasis, sample sizes were powered to be able to detect an upper 95% confidence interval (CI) threshold of 0.1% in children aged 5–9 years. Target grades chosen to represent the majority of pupils aged 5–9 years were grades 1–3 for public schools and grades 1–4 for private schools. From these target grades in the selected schools, pupils were randomly selected and enrolled in the survey. A total of 2500–2700 participants aged 5–9 years were included in each LGA (Table 3).

**Table 3:** Schools selected for inclusion in iTAS in the local government areas/implementation units

State	LGA (IU)	Total number of selected schools	Number of children planned to be tested
Cross River	Bekwarra	34	2590
Taraba	Gashaka	30	2500
	Karim Lamido	52	2700
Yobe	Bade	30	2700

### Diagnostic testing

Participants were tested using SD BIOLINE Oncho/LF IgG4 Biplex RDT (Ov16/Wb123) and the FTS. Dried blood spots were also taken and stored for additional testing. The Biplex RDT detects antibodies against both the *O*. *volvulus* Ov16 and the *W*. *bancrofti* Wb123 antigens. The FTS is an antigen test currently recommended by WHO for use as LF test.

### Data analysis

Frequencies and 95% CI for LF antigenemia and Wb123 and Ov16 positivity were determined using SPSS version 21 software (SPSS IBM Corp, Armonk, NY, USA). The 95% CI of the prevalence or frequencies was calculated by using a binomial CI exact test calculator.

## Results

### Pre-iTAS

#### Demographic characteristics of study participants during Pre-iTAS

A total of 5312 participants from 20 communities were enrolled in the study. Of these 5412 participants, 5244 (98%) were tested across the five LGAs (Table 4). Of the 2101 children aged 5–9 years who were tested in four communities in each LGA, 1052 (50%) were male. Of the 3143 individuals aged ≥ 10 years tested in a subset of two communities in each LGA, 1363 (43%) were male.

**Table 4:** Distribution of study participants by age and sex

S/N	State	LGA	Name of community	No. of children aged 5 to < 10 years sampled			No. of older children and adults aged 10 years sampled			Total no. of children sampled
				Male	Female	Total	Male	Female	Total	
1	Yobe	Bade	Dagona	54	46	100	99	211	310	410
2			Gafiwa	58	58	116	0	0	0	116
3			Tagali	54	51	105	153	158	311	416
4			Gwio Kura	47	58	105	0	0	0	105
Subtotal				213	213	426	252	369	621	1047
5	Cross River	Ikom	Ukpochi	47	55	102	0	0	0	102
6			Agbaragba	63	38	101	0	0	0	101
7			Etikpe	57	53	110	144	154	298	408
8			Balep	56	60	116	166	139	305	421
Subtotal				223	206	429	310	293	603	1032
9	Cross River	Bekwara	Nyanya-Ulim	49	52	101	0	0	0	101
10			Atibulum	49	51	100	0	0	0	100
11			Itekpa	46	55	101	73	215	288	389
12			Afrike-Okpeche	49	51	100	86	218	304	404
Subtotal				193	209	402	159	433	592	994
13	Taraba	Gashaka	Shinbon	29	32	61	0	0	0	61
14			Gashaka	47	46	93	0	0	0	93
15			Mayo-Selbe	115	81	196	249	102	351	547
16			Nyabar	38	32	70	160	221	381	451
Subtotal				229	191	420	409	323	732	1152
17	Taraba	K/Lamido	Bandawa	53	58	111	103	207	310	421
18			Panya O	51	52	103	130	165	295	398
19			Gwomu O	52	52	104	0	0	0	104
20			Kodei O	38	68	106	0	0	0	106
Subtotal				194	230	424	233	372	605	1029
Total				1052	1049	2101	1363	1780	3143	5244

#### LF antigenemia prevalence during Pre-iTAS

A total of 5191 valid tests was conducted using FTS. Based on the results of the valid tests, an overall antigenemia prevalence of 0.5% was recorded across all the study sites. Among the LGAs, the highest antigenemia prevalence of 1% (10/1026) was recorded in Ikom LGA in Cross River State, followed by Gashaka LGA (7/1152) in Taraba State and Bekwarra LGA (5/1,011) in Cross River State, with 0.6% prevalence recorded in both of the latter LGAs (Table 5). Among the communities surveyed, Agbaragba in Ikom LGA had the highest prevalence (5.9%; 6/101); no positive cases for LF, based on the FTS results, were recorded in Karim Lamido LGA. In total, there were 14 communities across all study areas where zero antigenemia-positive cases for LF using the FTS were recorded.

**Table 5:** LF prevalence by the Filariasis Test Strip, and seroprevalence of onchocerciasis (Ov16) and LF (Wb123) with the Biplex Rapid Diagnostic Test by age group

State	LGA	Community	Village type	Total no. valid FTS tests	No. of positive FTS results in children aged 5 to ≤ 10 years (%)	No. of positive FTS results in older children and adults aged ≥ 10 years (%)	Total no. positive FTS (%)	Total no. valid Biplex RDT	No. positive Biplex RDT in children aged 5 to ≤ 10 years (%)		No. positive Biplex RDT in children aged ≥ 10 years (%)		Total no. positive Biplex RDT results (%)	
									Onchocerciasis	LF	Onchocerciasis	LF	Onchocerciasis	LF
Yobe	Bade	Dagona		400	0	0	0	410	0	0	0	0	0	0
		Gafiwa	FL	116	0	ND	0	116	0	0	ND	ND	0	0
		Tagali		380	1 (0.3)	2 (0.5)	3 (0.8)	415	0	0	0	0	0	0
		Gwio Kura	FL	105	0	ND	0	105	0	0	ND	ND	0	0
Subtotal				1001	1 (0.1)	2 (0.2)	3 (0.3)	1046	0	0	0	0	0	0
Cross River	Ikom	Ukpochi	FL	102	0	ND	0	102	0	0	ND	ND	0	0
		Agbaragba	FL	101	6 (5.9)	ND	6 (5.9)	101	1 (1.0)	3 (3.0)	ND	ND	1 (1.0)	3 (3.0)
		Etikpe		405	0	0	0	408	2(0.5)	0	70 (17.2)	1 (0.3)	72 (17.7)	1 (0.3)
		Balep		418	0	4 (1.0)	4 (1.0)	421	1 (0.24)	0	68 (16.2)	0	69 (16.4)	0
Subtotal				1026	6 (0.6)	4 (0.4)	10 (1.0)	1032	4 (0.4)	3 (0.3)	138 (13.4)	1 (0.1)	142 (13.8)	4 (0.4)
Cross River	Bekwara	Nyanya-Ulim	FL	101	0	ND	0	100	4 (4)	0	ND	ND	4 (4)	0
		Atibulum	FL	100	0	ND	0	100	0	0	ND	ND	0	0
		Itekpa		407	2 (0.5)	4 (1.0)	6 (1.5)	407	0	0	27 (6.6)	4 (1.0)	27 (6.6)	4 (1.0)
		Afrike-Okpeche		403	0	0	0	404	0	0	95 (23.5)	15 (3.7)	95 (23.5)	15 (3.7)
Subtotal				1011	2 (0.2)	4 (0.4)	6 (0.6)	1011	4 (0.4)	0	122 (12.1)	19 (1.9)	126 (12.5)	19 (1.9)
Taraba	Gashaka	Shinbon	FL	61	0	ND	0	61	5 (8.2)	0	ND	ND	5 (8.2)	0
		Gashaka	FL	93	0	ND	0	93	0	3 (3.2)	ND	ND	0	3 (3.2)
		Mayo-Selbe		547	1 (2.1)	2 (0.4)	3 (0.6)	547	4 (0.7)	0	10 (1.8)	0	10 (1.8)	0
		Nyabar		451	0	4 (0.9)	4 (0.9)	451	3 (0.7)	0	31 (6.9)	3 (0.7)	34 (7.5)	3 (0.7)
Subtotal				1152	1 (0.1)	6 (0.5)	7 (0.6)	1152	12 (1.0)	3 (0.3)	41 (3.6)	3 (0.3)	49 (4.3)	6 (0.5)
Taraba	K/Lamido	Bandawa		395	0	0	0	421	0	0	0	2	0	2
		Panya O		396	0	0	0	398	0	0	0	0	0	0
		Gwomu O	FL	104	0	ND	0	104	0	0	ND	ND	0	0
		Kodei O	FL	106	0	ND	0	106	0	0	ND	ND	0	0
Subtotal				1001	0	0	0	1029	0	0	0	2 (0.2)	0	2 (0.2)

*LF* lymphatic filariasis, *FTS *Filariasis Test Strip (FTS; Alere Inc.),* LF* lymphatic filariasis, ND not determined,* Ov16/Wb123* filarial antigens,* RDT* rapid diagnostic test (SD BIOLINE Oncho/LF IgG4 Biplex RDT [Ov16/Wb123])

#### LF and onchocerciasis seroprevalence using the SD Bioline Biplex RDT during Pre-iTAS

Using the Biplex RDT, a total of 31 LF-seropositive cases (< 0.1%) among were detected from the valid tests in all the 20 communities visited for all ages. The highest number (19) was recorded in Bekwarra LGA in Cross River State, while Bade LGA in Yobe State had zero LF-seropositive cases. In only two communities were LF-seropositive cases (3 each) recorded in children aged < 10 years: Agbaragba community in Ikom LGA (3.0%) and Gashaka in Taraba State (3.2%). A total of 317 onchocerciasis-seropositive cases (6.0%) were detected from the valid tests using the Biplex RDT in all the 20 communities visited for all ages. The highest number (*n* = 142) was recorded in Ikom LGA, followed closely by Bekwarra LGA (126), both in Cross River State (Table 5). No LF-seropositive cases were recorded in Bade LGA in Yobe State and Karim Lamido in Taraba State. Onchocerciasis-seropositive cases, detected with the Biplex RDT, were detected in children aged < 10 years in seven communities, with the highest seroprevalence of 8.2% being recorded in Shibon community in Gashaka LGA, Taraba State.

**Fig. 3:**
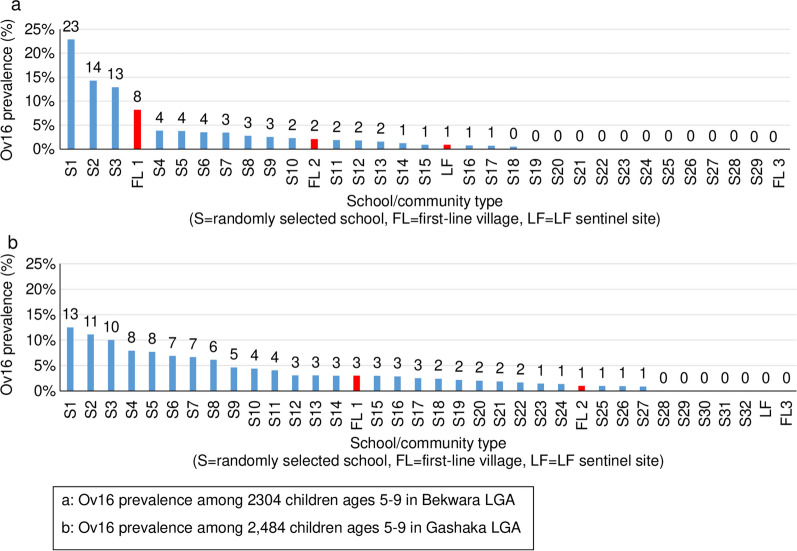
Prevalence of the Ov16 filarial antigen by first-line village, LF sentinel site or school-based cluster, among 2304 children aged 5–9 years in Bekwara LGA (**a**), and in 2484 children aged 5–9 years in Gashaka LGA (**b**), Nigeria, surveyed during pre-iTAS or iTAS in April 2017. Abbreviations: ITAS, Integrated transmission assessment survey; LF, lymphatic filariasis

Figure 3 shows the prevalence of the Ov16 filarial antigen in Bekwerra (Cross River) and Gashaka (Taraba).

### iTAS

The results of the Pre-iTAS in the studied LGAs indicated that most of the communities had an antigenemia prevalence of < 2%. However, the Ikom LGA did not proceed to/was not included in iTAS due to the 5.9% prevalence recorded among children, who are considered serological markers of new infection, in the Agbaragba community.

#### Demographic characteristics of study participants during iTAS

A total of 148 schools were visited and sampled across four LGAs. Of these, 131 (88.5%) were classified as rural and 17 (11.5%) as urban. All (100%) communities where these schools were located had conducted 17–21 onchocerciasis MDA rounds and five to seven LF MDA rounds. Total enrolment in the sampled schools within the targeted grades were 17,707 children (Table 6). The highest number of pupils (11,533) were tested in Gashaka LGA. Both genders were equally represented, with 5804 (50.3%) female pupils and 5729 (49.7%) male pupils.

**Table 6:** Prevalence of LF antigenemia by FTS, LF Wb123 antibody by Biplex RDT and onchocerciasis Ov16 antibody by Biplex RDT, by gender, grade, age group and district

LGA/IU	No. of pupils tested	Gender		Grade				Age group		
		Male	Female	1	2	3	4	Age < 5 years	Age 5–9 years	Age 10+ years
*Bade (30 schools sampled; 8593 students enrolled in target grades)*										
Number tested	2875	1387	1488	1028	1032	775	40	0	2388	487
Number FTS positive (%)	0 (0)	0 (0)	0 (0)	0 (0)	0 (0)	0 (0)	0 (0)	NA	0 (0)	0 (0)
Number Biplex-LF positive	1 (< 1)	1 (< 1)	0 (0)	1 (< 1)	0 (0)	0 (0)	0 (0)	NA	1 (< 1)	0 (0)
Number Biplex-oncho positive (%)	0 (0)	0 (0)	0 (0)	0 (0)	0 (0)	0 (0)	0 (0)	NA	0 (0)	0 (0)
B*ekwara (34 schools sampled; 3,078 students enrolled in target grades)*										
Number tested	2622	1343	1279	1141	680	721	80	0	2222	400
Number FTS positive (%)	5 (< 1)	3 (< 1)	2 (< 1)	3 (< 1)	0 (0)	2 (< 1)	0 (0)	NA	5 (< 1)	0 (0)
Number Biplex-LF positive (%)	14 (1)	10 (1)	4 (< 1)	6 (1)	3 (< 1)	5 (1)	0 (0)	NA	12 (1)	2 (1)
Number Biplex-oncho positive (%)	81 (3)	35 (2)	46 (4)	28 (2)	26 (4)	27 (4)	0 (0)	NA	67 (3)	14 (3.5)
*Gashaka (30 schools sampled; 3,026 students enrolled in target grades)*										
Number tested	3026	1561	1465	1325	855	846	0	0	2551	475
Number FTS positive (%)	7 (< 1)	4 (< 1)	3 (< 1)	3 (< 1)	1 (< 1)	3 (< 1)	NA	NA	6 (< 1)	1 (< 1)
Number Biplex-LF positive (%)	13 (< 1)	8 (1)	5 (< 1)	5 (< 1)	4 (< 1)	4 (1)	NA	NA	8 (< 1)	5 (1)
Number Biplex-oncho positive (%)	53 (2)	42 (3)	11 (1)	8 (1)	19 (2)	26 (3)	NA	NA	31 (1)	22 (5)
*Karim Lamido (52 schools sampled; 3,010 students enrolled in target grades)*										
Number tested	3010	1438	1572	1321	777	912	0	253	2241	516
Number FTS positive (%)	0 (0)	0 (0)	0 (0)	0 (0)	0 (0)	0 (0)	NA	0 (0)	0 (0)	0 (0)
Number Biplex-LF positive (%)	1 (< 1)	0 (0)	1 (< 1)	0 (0)	0 (0)	1 (< 1)	NA	0 (0)	1 (< 1)	0 (0)
Number Biplex-oncho positive (%)	1 (< 1)	0 (0)	1 (< 1)	1 (< 1)	0 (0)	0 (0)	NA	NA	NA	0 (0)

*NA* Data not available

#### LF antigenemia prevalence during iTAS

Twelve LF antigenemia-positive cases (0.1%) were recorded in all the four LGAs included in the iTAS, of which six (50%) were in pupils in Grade one, one (8.3%) in a pupil in Grade two and five (41.7%) in pupils in Grade three. No case was recorded in pupils in Grade four. LF antigenemia-positive cases were found in pupils of both sexes at about the same proportions. Gashaka and Bekwerra LGAs had antigenemia prevalence of 0.2% and 0.2%, respectively, while zero prevalence was observed in both Bade and Karim Lamido LGAs. Of the total 12 positive cases, 11 (91.7%) were in children aged < 10 years and one (8.3%) was in a child in the ≥ 10-year age group (Table 6).

#### LF and onchocerciasis seroprevalence using SD Bioline Biplex RDT during iTAS

A total of 29 LF-seropositive cases (0.3%) were recorded from the valid tests using the Biplex RDT in all of the 148 schools visited. Of these cases, 12 (41.4%) were in pupils in Grade one, seven (24.1%) were in pupils in Grade two, and 10 (34.5%) were in pupils in Grade three. No case was recorded in pupils in Grade four. The majority (19, 65.5%) of pupils who tested positive were male. Bekwerra and Gashaka LGAs had almost the same seroprevalence: 0.5% and 0.4%, with 14 and 13 cases, respectively. Both Bade and Karim Lamido LGAs had one case each, with a seroprevalence of 0.03%. Among the 148 schools sampled, 19 (12.8%) had pupils who tested positive (> 0%), with the highest site-specific prevalence of 7.9% observed in Bekwerra LGA. A total of 135 onchocerciasis-seropositive cases (1.2%) were recorded from the valid tests using the Biplex RDT in the four sites; of these, 77 (57%) were male pupils and 58 (43%) were female pupils. A total of 99 (73.3%) seropositive cases were recorded among children aged < 10 years and 36 (26.7%) among children aged ≥ 10 years. Children in Grade three had the highest number of positive cases (53, 39.3%) followed by those in Grade two (45, 33.33%) cases, and then children in Grade one (37 cases, 27.4%). No seropositive cases were recorded among children in Grade four. Of the four sites, the highest seroprevalence (3.1%, 81 cases) was recorded in Bekwerra LGA. Bade LGA had zero sero-prevalence. Only one positive case (0.003%) was observed in Karim Lamido LGA while 1.8% seroprevalence (53 cases) was recorded in Gashaka LGA. Sero-prevalence among those children aged < 10 years was 1.1%, with Bekwerra having 3% seroprevalence and Gashaka a seroprevalence 1.2% among this age group. Of the 148 schools sampled, onchocerciasis-positive cases were recorded in 46 (31.1%) schools, with a seroprevalence > 0.1%, with the highest site-specific prevalence of 22.9% recorded in Gashaka LGA. Of these 46 schools, 24 were located in Bekwerra LGA and 20 were located in Gashaka LGA. Five schools (3 in Gashaka LGA and 2 in Bekwerra LGA) had an onchocerciasis seroprevalence > 10% (Table 6).

## Discussion

Effective monitoring and evaluation are important components of MDA programmes throughout their life span. They are needed to effectively assess whether the infection being addressed has been reduced to levels at which both transmission may be assumed to be no longer sustainable and recrudescence is unlikely to occur even in the absence of drug intervention [15]. Surveillance should target the entire population in an EU or IU. However, due to limited resources and time constraints, a careful selection of sites and consideration of the most vulnerable population to be monitored are needed. The preliminary results from the iTAS conducted with FTS indicate an overall LF antigenemia prevalence of 0.1% with slight insignificant variations among the IUs. Bade and Karim-Lamido LGAs had zero antigenemia prevalence, while Bekwara and Gashaka LGAs had 0.2%. However, in Gashaka LGA, antigenemia prevalence was above 2%. The findings are consistent with the results obtained from the pre-iTAS (with the exception of those on onchocerciasis seroprevalence from Bekwerra and Gashaka LGAs). Interestingly, the pre-iTAS LF results from Bekwerra LGA, which were close to the 2% seroprevalence criteria to qualify for the iTAS, improved in the subsequent iTAS, for which 0.2% seroprevalence was recorded, thus justifying the decision to include this LGA in the iTAS. This result may suggest that for LF, pre-iTAS LGAs for which the results are either below or above the 2% antigenemia prevalence cut-off should be considered for inclusion in the iTAS. However, the observed prevalence for each of the two sites evaluated during the pre-iTAS in Bekwerra LGA had a wider margin (i.e. sentinel 4 [1%] vs spot-check 15 [3.7%] when compared with other IUs). This result suggests that the transmission intensity varies between communities within an IU, irrespective of whether the aggregated estimate is 1.9%. It is therefore important that actions taken within a programme are tailored towards disaggregated estimates, as aggregated estimates may mask disease distribution patterns and, hence, mislead programmatic decision-making [16].

Nevertheless, based on the WHO guidelines [4], four LGAs were deemed to have passed TAS for LF. However, in IUs where prevalence exceeds 2%, targeted MDA would be implemented irrespective of the outcomes of this iTAS. Subject to the availability of resources, however, it will be suitable to conduct additional treatment campaigns targeted at the ‘hotspot’ schools and to further conduct tests following the second round of treatment to determine whether transmission is ongoing.

The Wb123/Ov16 Biplex RDT results suggest that transmission of onchocerciasis infection may have been interrupted in Bade and Karim Lamido LGAs, given the zero seroprevalence recorded during the iTAS. This result is further corroborated to a large extent by similar results from the pre-iTAS. Nevertheless, these results are not sufficient to recommend a stop/halt for onchocerciasis MDA in any of these LGAs for two major reasons: (i) type of test use and (ii) the unit of evaluation. Firstly, the biplex serological test used is not yet approved as a tool for surveillance. Similarly, the Ov16 RDT serological test, which has been approved for surveillance, is not to be solely utilized for making decisions in whether or not to stop onchocerciasis MDA. As such, we collected dried blood smears for future laboratory testing with an Ov16 enzyme-linked immunosorbent assay (ELISA) or other test that may be available in the future. Secondly, the decision to stop MDA for onchocerciasis is made at the level of transmission zones, which vary by country, unlike IUs for LF. This has been previously demonstrated in the states of Plateau and Nassarawa in Nigeria. In Nigeria, the National Onchocerciasis Elimination Committee, an advisory body to the government, has decided to use the state as the evaluation unit for the stop-MDA decision, taking into consideration the large expanse of overlapping transmission sites across the IUs. As such, the decision to stop onchocerciasis MDA will require a state-wide assessment followed by entomological evaluation of adult black flies to determine < ½000 infectivity rate before pronouncing any stop of the MDA [5, 17].

The current status of LF recorded in the studied LGAs was expected given the treatment coverage of > 85% achieved annually, which had initially qualified these LGAs for inclusion in the Pre-iTAS, the low pre-intervention antigenemia prevalence of < 10%, with three of the five LGAs studied having ≤ 2.0% antigenemia prevalence, and the long history of treatment with ivermectin, ranging between 17 and 21 years. This result agrees with pre-existing notions on the possibility of an indirect or overlapping microfilaricidal effect of ivermectin MDA on LF transmission [18].

The relatively high level of transmission of onchocerciasis observed in Gashaka raises concern. Although the pre-intervention endemicity and outcomes of previous epidemiological assessments using skin snips had been high [19], the impact of mass treatment over the years ought to have been observed in the pattern of endemicity. Similarly, the relatively high transmission of onchocerciasis in Bekwerra LGA was unexpected due to the low pre-intervention endemicity and the long history of annual treatments in this LGA. It is noticeable that transmission remains ongoing in both Bekwerra and Gashaka LGAs based on the level of seroprevalence recorded among children, who are serological markers of a new infection. These may be due to inadequate compliance to (attrition, fatigue) annual treatment by the target populations in those areas. Consequently, strengthening of MDA interventions and the supervision process is of critical importance [16].

The Ov16 results from both the pre-iTAS and iTAS revealed a number of important issues. Results for Ov16 during the iTAS indicated that prevalence increased with age. Given this outcome, a sampling strategy that includes children aged 5–9 years compared to those aged 6–7 years, as in traditional LF TAS, resulted in a more conservative pre-stopping assessment. WHO may therefore reconsider the justification to include children in this age bracket in future assessments. The pre-iTAS results indicated that purposeful selection of first-line villages was insufficient at identifying the sites with the highest Ov16 prevalence by RDT in children aged 5–9 years, suggesting that random sampling may be necessary to identify sites with the potential transmission. However, in employing random sampling there may be a need to exclude from the sampling frame all villages with known information or a high likelihood of being in very low endemic areas based on latest entomological and/or epidemiological assessments. It has been advised that exclusion mapping be done before selection of villages for assessment [20].

## Conclusion

This study provides additional information on the current burden of onchocerciasis and LF in the four IUs sampled and identified that LF-MDA can be safely stopped in all IUs, although an additional round of MDA in schools where antigenemia prevalence is > 2% is recommended. MDA for onchocerciasis needs to continue, although this may pose a challenge for LF surveillance. Xeno-monitoring of blackflies in LGAs where the threshold for onchocerciasis elimination by serological assessment has been met in the context of the Operational Research OR study sites is recommended, followed by strengthening of onchocerciasis MDA and improved supportive supervision, particularly in Bekwerra and Gashaka LGAs. Based on the preliminary results from all four sites, this study has fulfilled the primary objective of determining the programmatic feasibility of an iTAS to be used to simultaneously assess onchocerciasis and LF prevalence in areas co-endemic for the two infections that have completed the recommended treatment for one or both infections, and to make decisions on how to proceed.

## Data Availability

All data will be made available to the journal.
